# Supplementary material for: A Theranostic Nanocomplex Combining with Magnetic Hyperthermia for Enhanced Accumulation and Efficacy of pH-Triggering Polymeric Cisplatin(IV) Prodrugs
Source: Pharmaceuticals (Basel). 2022 Apr 14;15(4):480. doi: 10.3390/ph15040480 (PMC9025582; doi:10.3390/ph15040480)
Supplement: Supplementary file 1 [file pharmaceuticals-15-00480-s001.zip › pharmaceuticals-1620884-supplementary.pdf]

Supplementary Materials

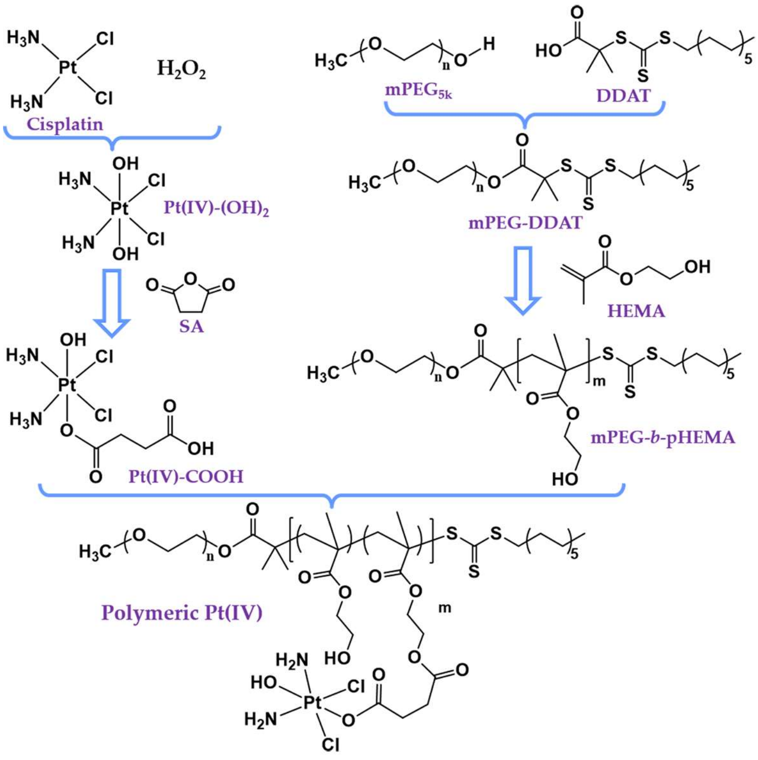

Figure S1: Synthetic scheme of polymeric Pt(IV)

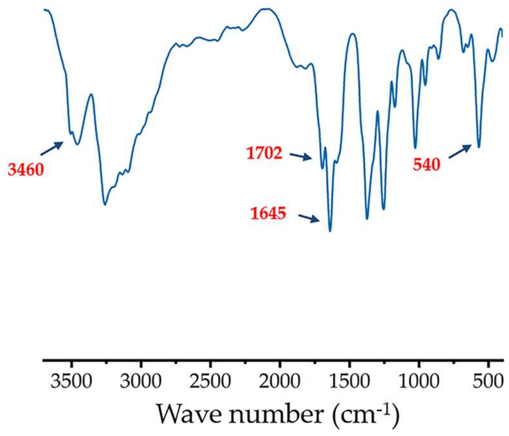

Figure S2: FTIR spectrum of Pt(IV)-COOH

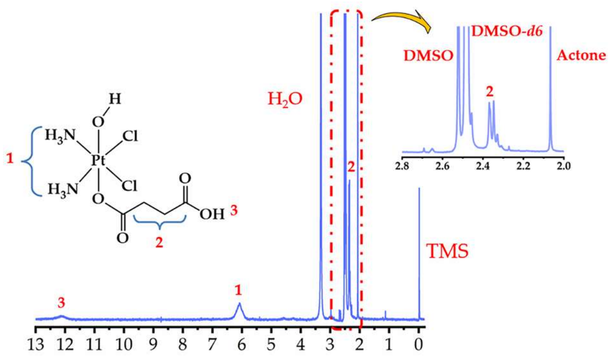

Figure S3: ^{1}H NMR spectrum of Pt(IV)-COOH

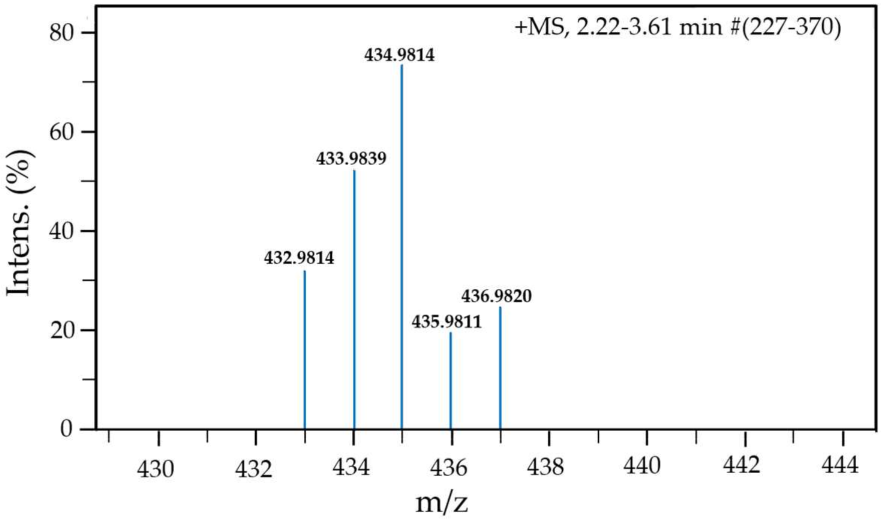

Figure S4: Mass spectrum of Pt(IV)-COOH

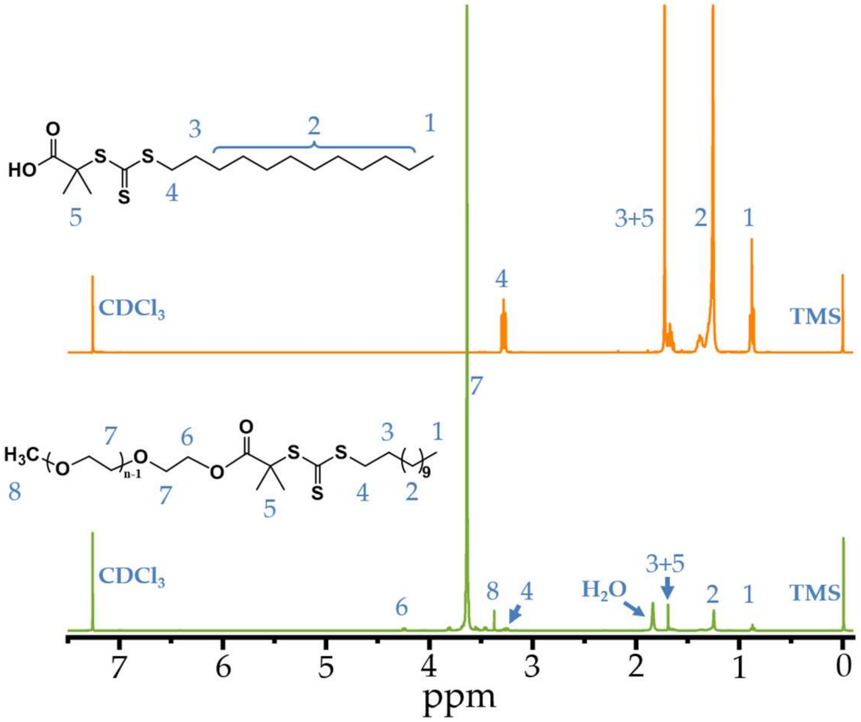

Figure S5: ^{1}H NMR spectra of DDAT and mPEG-DDAT

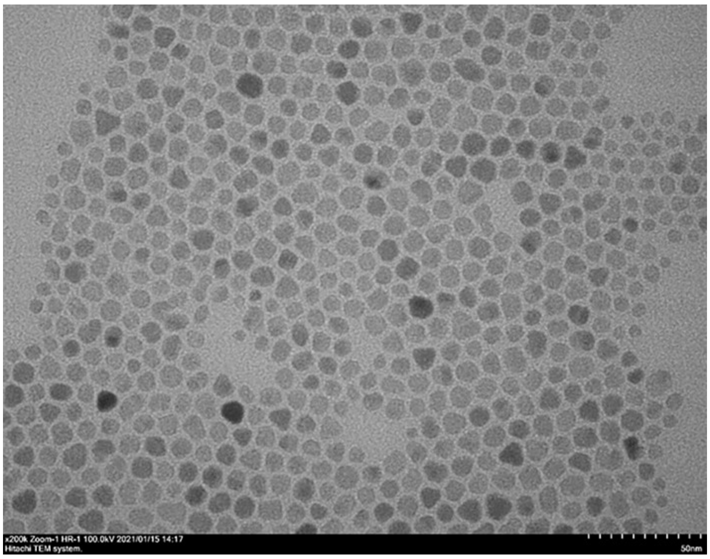

Figure S6: TEM result of monodispersed $Mn_{0.6}Zn_{0.4}Fe_2O_4$ nanoparticles

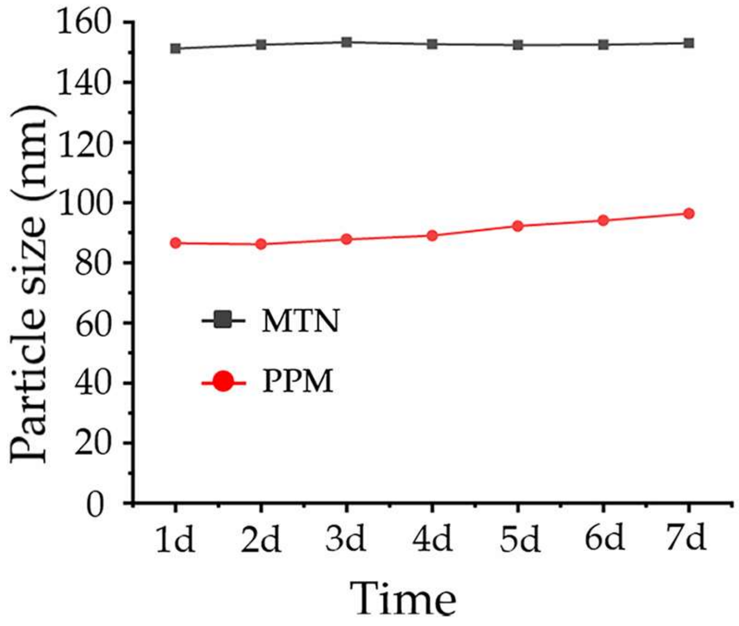

Figure S7: The time-dependent hydrodynamic diameter of PPM and MTN in PBS

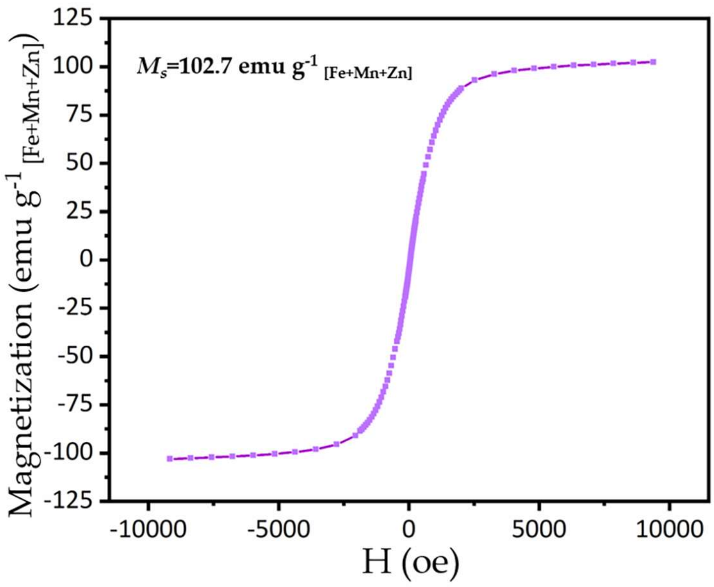

Figure S8: Magnetization curve of the Mn0.6Zn0.4Fe2O4 at 300 K

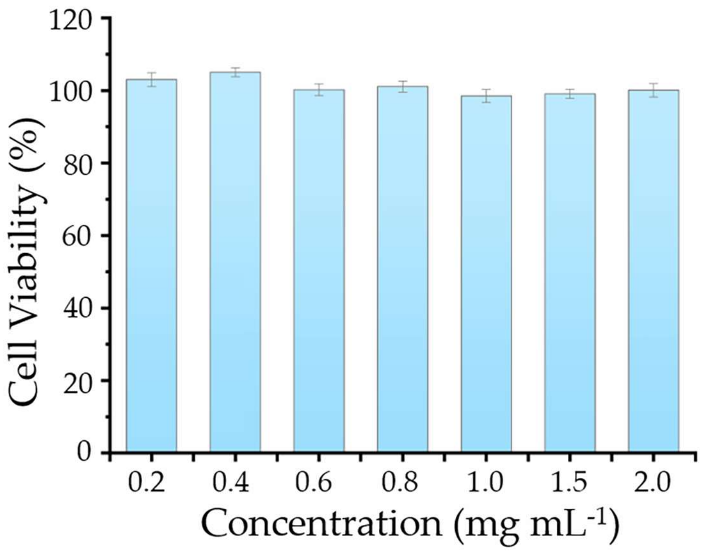

Figure S9: The biocompatibility of mPEG-b-pHEMA
